# Pre-clerkship Teaching and Learning in the Virtual Learning Environment: Lessons Learned and Future Directions

**DOI:** 10.1007/s40670-022-01694-8

**Published:** 2022-11-19

**Authors:** Justin L. Sewell, Mihir Joshi, Cassandra Thanh, Chantilly Apollon, Elizabeth Austin, Christian Burke, Susannah Cornes, John A. Davis, Michelle Hermiston

**Affiliations:** 1 Department of Medicine, Division of Gastroenterology, University of California San Francisco, San Francisco, CA USA; 2 School of Medicine, University of California San Francisco, San Francisco, CA USA; 3 Foundation for California Community Colleges, Sacramento, CA USA; 4 Department of Neurology, University of California San Francisco, San Francisco, CA USA; 5 Department of Medicine, Division of Infectious Disease, University of California San Francisco, San Francisco, CA USA; 6 Department of Pediatrics, Division of Oncology, University of California San Francisco, San Francisco, CA USA

**Keywords:** Virtual learning, Sociocultural learning theory, Cognitive load theory, Undergraduate medical education, Curriculum development

## Abstract

In response to the COVID-19 pandemic, we developed and implemented a theory-informed process to adapt a comprehensive pre-clerkship medical school curriculum to run in the virtual learning environment utilizing sociocultural learning theory and cognitive load theory. Of 124 student respondents, 45% rated virtual learning as very or extremely effective, and 49% as moderately effective. Positive aspects of virtual learning included effectiveness of chat moderators, displaying pronouns on Zoom, active learning technology, and captioning and transcription. Negative aspects included access to technology and feeling isolated from community. Overall course ratings, examination performance, and work hours did not differ pre- and post-implementation.

## Background

As the COVID-19 pandemic progressed, medical education leaders rapidly overhauled curricula to promote learning and milestone achievement while also ensuring student, faculty, staff, and patient safety [1]. Pre-clerkship curricula were largely transitioned to “virtual learning environments” (VLEs). At the University of California San Francisco School of Medicine (UCSF-SOM), we developed and implemented a rigorous process to meet these challenges. Whereas others have reported on curricular challenges and responses broadly [2], we describe in this Short Communication our local efforts to adapt a highly condensed 18-month pre-clerkship curriculum to run largely in the VLE and its impact on student satisfaction, performance, and work hours. By considering our experience, including principles we developed, approaches we took, and lessons we learned, we hope to contribute to scholarly discussion of how to promote effective pre-clerkship medical education, regardless of delivery platform.

## Activity

### Process and Principles

**Fig. 1:**
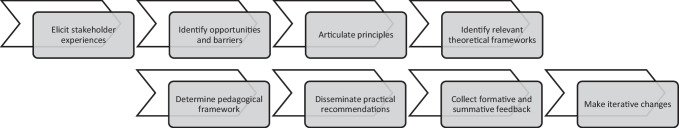
Virtual learning environment planning process

A workgroup of eight faculty, four staff, six students, one resident, and two technology stewards generated recommendations to promote effective, engaging distance learning that maintained a positive learner experience, promoted community development and professional identity formation, and maintained respect for personal boundaries. The workgroup developed and followed a rigorous process (Fig. 1) framed around pedagogy, student and faculty development, and social experience and community development and inductively developed guiding principles (Table 1). We identified sociocultural learning theory [3] as salient to every principle and also at greatest risk in the VLE setting. We also considered the VLE as a significant threat to cognitive learning; through the lens of cognitive load theory [4], we hypothesized that aspects of remote learning might lead to greater extraneous load, which could compromise learning. For example, teacher and student lack of familiarity and facility with remote learning tools and the need to learn in isolation through screens rather than real-life interactions were considered potential sources of extraneous load.

**Table 1:** Principles guiding virtual learning environment teaching and learning

Principle	Explanation
Equity	Learning sessions should be designed with student differences in background, ability, and individual experiences in mind. We should explicitly work to design and standardize opportunities for success that are not impacted by these differences. Specific potential threats to equity that should be mitigated include: variability in small group instructors, processes and learning environment, and disparities in access to technology
Engagement	In order to promote self-regulated learning, student engagement with learning processes must be promoted. Threats to engagement include Zoom fatigue, the lack of active learning, preparedness of faculty for virtual learning platforms, and the absence of social context and “normal” interactions among students and between students and faculty. Engagement with the curriculum is critical to promote germane cognitive load, which leads to learning. Flexibility to accommodate different learning styles and variability in session design are deemed central to enhancing student engagement
Effectiveness	Effective instruction, facilitation, and learning are always critical in a complex and fast-paced curriculum. Complexity of material must be matched to learners’ prior experience and scaffolded appropriately. Distractions and other sources of extraneous cognitive load must be minimized. Students must be provided with strategies to promote learning. These demands were challenging to meet even before the COVID-19 pandemic, yet they are even more challenging in the virtual learning environment
Communities of practice	Sociocultural theories of learning emphasize that learning occurs not in a cognitive vacuum, but within learning environments as learners interact with teachers, fellow learners, and other stakeholders. COVID-19 impacts our ability to engage and interact with others in ways that feel “normal” and that support development and growth of communities of practice
Professional identity formation	Educators have a duty to promote development of professionals to practice in the field of medicine. This requires not only cognitive learning, but also professional identity formation. Related to communities of practice, COVID-19 may impact when, where, and how students interact with one another, with faculty and staff, and with patients (actual or standardized). It is critical to make distance learning feel like an authentic progression of learning to be a doctor including responsibility for life long-learning and engaging with patients in telemedicine settings
Wellness and fun	Learning medicine is among the most challenging tasks a learner can undertake, but it should also provide space and time for activities that promote wellness and should, whenever possible, be enjoyable for learners. These principles should guide the format of the curriculum and design of each teaching session
Work hours and boundaries	In addition to monitoring work hours, we must consider that the virtual learning environment poses threats to boundaries. Whereas formal classwork could formerly be accomplished entirely on campus, students in the VLE must learn almost exclusively from their homes. We aim to reduce intrusiveness of the curriculum into students’ personal spaces and to maintain appropriate work hours and boundaries

### Curricular Adaptation

*Large group didactics* previously offered in the classroom were transitioned to synchronous Zoom sessions. A faculty member served as “chat moderator” to address student questions directly or to pose common questions to the lecturer. Lecturers were encouraged to use active learning tools such as Zoom Polls. Providing real-time closed captioning during sessions and video recordings immediately after sessions promoted equity and effectiveness. Lecturers were encouraged to introduce themselves fully, aiming to make visible their humanness. *Small group discussions* were held over Zoom. Facilitators were encouraged to introduce themselves fully and underwent rigorous standardized training in technology and distance learning pedagogy. To promote student engagement, we incorporated synchronous editing technology such as Google Docs. In some sessions, students were pre-assigned to specific roles to promote professional identity formation. We allotted extra time for checking in and checking out and encouraged students to keep their video on and to promote communities of practice; however, to respect personal boundaries, students were not required to turn their video on. *Clinical skills and quality improvement sessions* were maintained in-person with accommodations to align with public health guidelines and with changes to promote VLE principles that posed some of the greatest challenges: communities of practice, professional identity, and wellness and fun. A small number of sessions were held over Zoom when it served the learning objectives, for example, telehealth training. *Anatomy labs* transitioned from small group student cadaver dissections to a combination of large and small group sessions. A faculty member demonstrated live synchronous prosection; this provided equitable experience for all students and was intended to promote effective and engaging learning. Faculty quizzed students using Zoom Polls during the demonstration, and chat moderators addressed student questions. *Examinations*, previously held in computer labs, were administered remotely. Students attested understanding that examinations were closed-book and agreed to follow the honor code.

### Technology Implementation

In addition to the above, we established 70 standardized Zoom accounts for curricular sessions. Each small group had its own Zoom account, and each student class had a single Zoom account for lectures; this promoted consistency, equity, and effectiveness. Students were provided with personal Zoom accounts for school and personal purposes.

### Faculty, Staff, and Student Development

The workgroup communicated with stakeholders via meetings and a formal report. Stakeholders were oriented to VLE principles and were given VLE skill-building sessions addressing Zoom essentials, polling, breakout rooms, engagement, collaboration, and humanizing the VLE. We trained volunteer student leaders to serve as technology stewards during small group sessions.

### Assessment

We compared assessment data from course evaluations and examination performance for VLE and pre-VLE periods. Examinations included open-ended questions that were graded as “meets expectations,” “borderline meets expectations,” or “does not meet expectations” using rubrics. Examination content, grading practices, and standard for passing (70% meets expectations) did not change. Consistent with our principles (Table 1), we analyzed work hours (self-reported by students in course evaluations as per usual practices). Other data were collected via surveys and focus groups. Qualitative data were subjected to content analysis.

## Results and Discussion

### Student Perspectives

In a survey administered September 2020, 45% of 124 student respondents reported virtual learning was very or extremely effective, and 49% indicated it was moderately effective. Only 52% indicated they enjoyed virtual learning (64% of MS2 class, 44% of MS1 class), and 68% of MS2’s reported virtual learning was somewhat or much worse than in-person learning. Content analysis revealed both successes and challenges. Successes included effectiveness of chat moderators, displaying pronouns on Zoom, active learning technology tools, and captioning and transcription. Challenges included inadequate access to technology and bandwidth, limited social and community-building activities, and “Zoom fatigue.”

### Course Ratings

Overall course ratings were similar comparing in-person learning (seven courses, average 4.14, 1–5 scale) and VLE learning (seven courses, average 4.12, 1–5 scale).

### Examination Performance

Examination performance was similar comparing in-person learning (97.8% pass rate out of 2177 student examinations) and during VLE learning (97.9% pass rate out of 2426 student examinations). There were no incidents of unethical examination behavior.

### Work Hours

Weekly work hours were similar comparing in-person learning (seven courses, average 48.9 h per week) and virtual learning (seven courses, average 49.7 h per week).

### Adoption of VLE Principles

The VLE principles our working group developed were formally integrated into the standard annual course evaluation process.

### Discussion

The COVID-19 pandemic stimulated rapid change in medical school curricula. At the UCSF-SOM, we followed a rigorous, theory-informed process to promote learning during this time. While objective measures of students’ learning and work hours did not change, student perceptions of learning and enjoyment declined during the VLE. Taken within the context of our single-site experience, lessons learned have implications for pre-clerkship medical school curricula.

We identified sociocultural learning theory and cognitive load theory as relevant to VLE principles, yet sociocultural aspects of learning appeared more greatly impacted than cognitive learning. Barely half of students indicated they enjoyed learning in the VLE, and narrative comments suggested deficits in socioculturally oriented principles of professional identity formation, communities of practice, and wellness and fun. Curricula often focus on professional identity formation but leave tacit responsibility for the latter two principles to students. Our experience suggests schools ought to assume intentional roles in promoting community formation and wellness.

One positive sociocultural impact was instructors’ intentional efforts to make themselves known and available to students and their positioning of “being in this together” with students. We argue it is critical that faculty and staff engage with students and their communities to promote wellness and professional identity formation, as opposed to a stereotypical hierarchical approach.

Despite potentially high levels of extraneous load contributed by virtual learning and fears about pandemic, student performance on examinations did not diminish in the VLE. Likewise, course ratings suggested similar high quality of teaching. These findings suggest cognitive learning was maintained in the VLE, despite logistical changes.

In 2020, Emanuel wrote that the COVID-19 pandemic would spell the demise of classroom-based pre-clerkship instruction [5] and that schools would transition to exclusively online preclinical training. The commentary referred to in-person lectures as “a waste of everyone’s time.” The commentary hewed predominantly to a cognitive framework (minimally affected in our experience) and did not refer to sociocultural aspects of learning (highly affected in our experience). The commentary also did not mention “Zoom fatigue” (which was the single most commonly cited grievance of students and faculty alike) and did not address inequitable impacts on less privileged students entering medical school with less understanding of the culture of medicine[6], as well as those with learning disabilities or with limited access to technology. In contrast, our experience suggests that in-person pre-clerkship learning is of vital importance, particularly to support sociocultural learning and professional identity formation.

Our experience suggests virtual learning has potential advantages when used strategically. The ease and availability of virtual meeting platforms permits schools lacking local expertise to recruit faculty from other institutions to teach, benefitting student learning and faculty development alike. Strategies we “discovered” during the VLE experience, such as polling software, may help students remain engaged and promote learning during didactic sessions. The chat moderator role allows students to ask more detailed questions and better engage with the material. Encouraging faculty to present themselves authentically and providing dedicated time and space for students and faculty to get to know each other can promote professional identity and community formation. Finally, and perhaps of greatest future impact, schools can maintain the nimble and flexible approach required by the pandemic to continue improving curricula and combat the structural inertia present in many medical schools.

While the pandemic continues to wax and wane, we know that future novel challenges yet await. Our local experience supports a nimble, theory-informed approach engaging stakeholders across disciplines and levels of training in order to promote successful curricular adaptations in times of crisis, thereby promoting optimal medical education for our learners, teachers, and patients.

## Data Availability

Not applicable.
